# An Interesting Case of Congenital Intrahepatic Porto-hepatic Shunt as a Cause of Unexplained Encephalopathy

**DOI:** 10.7759/cureus.7639

**Published:** 2020-04-11

**Authors:** Arsalan A Alvi, Jose Pichardo, Sonali Gupta, Pradeep Goyal, Joseph Mattana

**Affiliations:** 1 Internal Medicine, St. Vincent's Medical Center, Bridgeport, USA; 2 Internal Medicine, Frank H. Netter MD School of Medicine, North Haven, USA; 3 Radiology, University of Rochester Medical Center, Rochester, USA

**Keywords:** congenital, intrahepatic, porto-hepatic shunt, encephalopathy, ammonia, extra-hepatic, anomaly, hepatic, cirrhosis, lactulose

## Abstract

Congenital portosystemic shunts can be divided into two types: intrahepatic shunts in which there is an abnormal connection between the branches of the portal vein and either the inferior vena cava or the hepatic veins and less commonly the extrahepatic type in which the portal system is connected to one of the branches of the mesenteric veins. Here we describe a 73-year-old woman who was admitted to the hospital with clinical evidence of encephalopathy and was found to have hyperammonemia. Abdominal computed tomography angiography was performed and revealed a dilated portal vein measuring up to 1.8 cm at the porta-hepatis along with dilated superior mesenteric and splenic veins. Multiple dilated vascular channels were identified within the right hepatic lobe. An intrahepatic portosystemic shunt between an enlarged middle hepatic vein and two separate branches of the right portal vein was demonstrated. A liver biopsy showed normal architecture with no evidence of inflammation or fibrosis. Portosystemic shunts are rare and often detected in adulthood but should be considered as an important cause of unexplained encephalopathy in the absence of cirrhotic liver disease or hepatic trauma. Given that the size of such shunts increases with age, older persons are more prone to the effect of toxic metabolites.This age-associated increase in shunt size may help explain why some patients remain asymptomatic until later in their life which may account for the late presentation in our patient.

## Introduction

Congenital portosystemic shunts can be divided into two types: intrahepatic shunts in which there is an abnormal connection between the branches of the portal vein and either the inferior vena cava or the hepatic veins and less commonly the extrahepatic type in which the portal system is connected by one of the branches of the mesenteric veins. Congenital intrahepatic portosystemic shunts or Abernethy malformations are rare anatomical abnormalities characterized by an abnormal connection between the portal vein and the hepatic vein [1,2]. Intrahepatic shunt anomalies occur predominantly in males and have been reported to affect only 0.0235% of the population [3,4]. Here we describe a case of a non-cirrhotic woman with recurrent encephalopathy who was diagnosed at age 73 with a congenital intrahepatic type II Abernethy malformation.

## Case presentation

A 73-year-old woman with rheumatoid arthritis and Gilbert syndrome was admitted to the hospital with clinical evidence of encephalopathy. On presentation her temperature was 36.5° C, blood pressure 165/73 mmHg, pulse rate 76 bpm, and respiratory rate 16 breaths/minute. Her physical examination was otherwise notable for encephalopathy in which she was initially agitated and combative followed by lethargy and disorientation to time, place, and person. There were no focal neurological deficits nor stigmata suggestive of central nervous system infection, vasculitis or cirrhosis. Laboratory investigations included a leukocyte count of 4900 cell/mm3 (normal: 4.8-10.8 * 10^3^cells/mm^3^), hemoglobin 12.2 g/dL (14.0-18.0 g/dl), platelet count 339,000/mm^3^ (140,000-440,000/mm^3^), prothrombin time (PT) 11.5 sec (9.4-11.7), international normalized ratio (INR) 1.03 (0.93-1.11), sodium 144 mmol/L (136-145 mmol/L), potassium 4.0 mmol/L (3.5-5.1 mmol/L), chloride 113 mmol/L (98-111 mmol/L), bicarbonate 25 mmol/L (21-31 mmol/L), blood urea nitrogen (BUN) 10 mg/dL (6-20 mg/dl), creatinine 0.5 mg/dL (0.6-1.2 mg/dl), calcium 9.6 mg/dL (8.6-10.0 mg/dl), albumin 3.8 g/dL (3.4- 4.8 g/dl), total protein 6.4 g/dL (6.4-8.3 g/dl), aspartate aminotransferase 20 U/L (8-20 U/L), alanine aminotransferase 15 U/L (10-40 U/L), alkaline phosphatase 68 U/L (25-100 U/L), total bilirubin 1.8 mg/dL (0.3-1.2 mg/dl), direct bilirubin 0.44 mg/dL (0.0-0.19 mg/dL) and ammonia level 165 mcmol/L (19-60 mcmol/L). Urinalysis and blood cultures were negative.

CT of the brain was unremarkable. Further laboratory testing revealed a negative human immunodeficiency virus (HIV) screen, hepatitis panel, and anti-smooth muscle antibodies. Abdominal ultrasonography revealed normal size and echotexture of the liver along with a dilated portal vein and dilated vascular channel within the right hepatic lobe. The liver appeared non-cirrhotic and a liver elastography showed a Metavir score of F1 indicating no evidence of cirrhosis.

**Figure 1:**
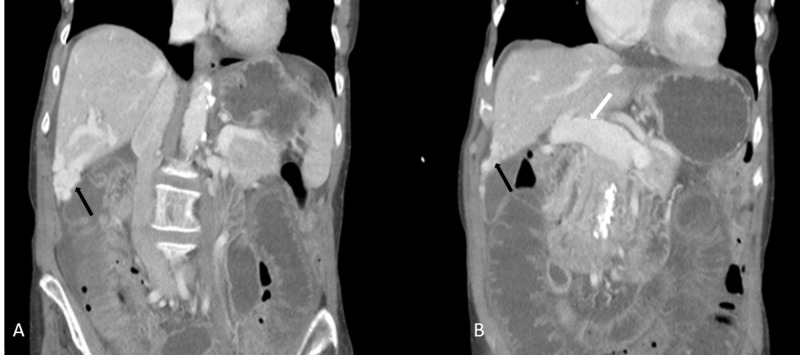
Coronal images of contrast-enhanced CT of the abdomen (A) A coronal view showing multiple dilated vascular channels (black arrow) in the right hepatic lobe (segment V). (B) Another coronal view which also shows dilated portal vein measuring up to 1.8 cm at the porta hepatis (white arrow) and multiple dilated vascular channels (black arrow) in the right hepatic lobe (segment V).

**Figure 2:**
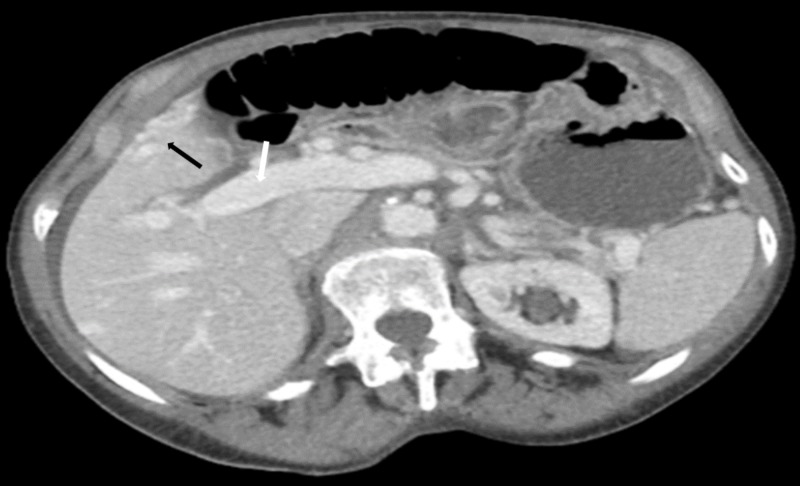
Cross-sectional view of CT scan of the abdomen and pelvis Cross-sectional view showing dilated portal vein measuring up to 1.8 cm at the porta-hepatis (white arrow) and multiple dilated vascular channels (black arrow) in the right hepatic lobe (segment V). An intrahepatic portosystemic shunt between the segment V portal venous branches and a peripheral hepatic vein draining into the middle hepatic vein was visualized (not shown here).

Abdominal CT angiography revealed a dilated portal vein measuring up to 1.8 cm at the porta-hepatis along with dilated superior mesenteric and splenic veins. Multiple dilated vascular channels were seen within the right hepatic lobe. An intrahepatic portosystemic shunt between an enlarged middle hepatic vein and two separate branches of the right portal vein was visualized (Figures 1-2).

A liver biopsy showed normal architecture with no sign of inflammation or fibrosis. She received conservative management with lactulose and rifaximin. Hyperammonemia and encephalopathy resolved after the second day of admission and she was discharged with close outpatient follow up by gastroenterology and interventional radiology.

## Discussion

This case was also presented at the ACG meeting in San Antonio in 2019 for the clinical vignette portion of the conference poster presentation [5].

Encephalopathy associated with portal-systemic shunt bypass without intrinsic hepatocellular disease is often referred to as Type B hepatic encephalopathy as illustrated in our case with ammonia being the mediator of the neuropsychiatric manifestations. Under normal conditions, ammonia is metabolized to urea by the urea cycle in the liver but in the presence of a portosystemic shunt, ammonia bypasses hepatic metabolism and entering the systemic circulation and further transitioning through the blood-brain barrier [6].

Portosystemic shunts are rare and often detected in adulthood but should be considered as an important cause of unexplained encephalopathy in the absence of cirrhosis [3]. It is well known that the size of such shunts increases with age making older people are exposed to the higher level of the toxic metabolite, resulting in confusion, lethargy or even frank encephalopathy [7]. This age-associated increase in shunt size may help explain why some patients remain asymptomatic until later decades in their life.

The embryological development of the hepatic system takes place between the fourth and the fifth week of organogenesis. During this period the portal vein develops from the vitelline system while the hepatic veins develop from the umbilical system which further divides into intrahepatic sinusoids. At week 5 of gestation, the vitelline veins should normally close. Failure of this process leads to the formation of a congenital intrahepatic portosystemic shunt [8]. Patients typically do not have any liver enzyme derangements and testing for intrinsic liver disease is usually negative. This condition can therefore be mistaken for a psychiatric disorder such as depression or psychosis or with dementia, especially in older people [9-10]. Most often the only positive finding is hyperammonemia which is crucial in guiding the clinician to obtain further diagnostic imaging in order to make the appropriate diagnosis [9]. Identification of affected patients is limited by the fact that only 35% of patients will have a marked ammonia level elevation and only 15% experience hepatic encephalopathy [11].

There are four subtypes of intrahepatic shunts. The most common is type 1 in which a solitary large vessel connects the right portal vein with the inferior vena cava [12]. The second most common is type 2 in which the distal part of the portal vein abnormally joins branches of the hepatic vein specifically within one hepatic lobe. In type 3 the shunt is arising from a congenital aneurysm between the peripheral branches of the portal vein and the hepatic vein. Finally, in type 4 multiple anastomoses occur between branches of both venous systems involving both hepatic lobes [13]. 

Similarly, extrahepatic shunts are divided into two subtypes based on the Morgan and Superina classification. In type 1, the intrahepatic segment of the portal vein is completely absent leading to the drainage of entire portal blood into the systemic venous system. Extrahepatic type 1 is further divided into subtypes 1a and 1b. In subtype 1a, the superior mesenteric and splenic veins drain separately into the inferior vena cava, while in subtype 1b, the superior mesenteric vein combines with splenic vein before draining into the inferior vena cava. In contrast, with type 2 extrahepatic shunt, the connection with the systemic circulation occurs via collateral veins of a partially patent portal vein. Type 2 extrahepatic shunts have a better prognosis as only some of the blood from the portal circulation drain into the systemic circulation [14-15].

Despite the type, portosystemic shunts are typically found incidentally. However, they may be associated with other congenital abnormalities such as duodenal atresia, absence of the ductus venosus, shunts associated with other organs, etc. Clinical presentation depends on the degree and the type of shunt, ranging from an incidental finding on imaging to hepatic encephalopathy [15].

There are many complications associated with these anomalies including pulmonary congestion leading to pulmonary hypertension in which patients will usually present with gradual shortness of breath, hepatorenal syndrome characterized by gradual worsening of renal function and signs of fluid retention, and acute manifestations similar to the case of our patient presenting with altered sensorium and hepatic encephalopathy. The prognosis of the disease depends on the extent of cardiac, renal and hepatic complications. Patients such as ours in which the condition is limited to only the hepatic system have a better prognosis [2].

Treatment modalities include an appropriate bowel regimen with the titration of lactulose, non-absorbable antibiotics, endovascular shunt closure or liver transplant [10-11]. The best treatment option depends on multiple factors such as the type and size of the shunt, symptoms, complications and other comorbidities. The severity of the shunt can be measured based on the portosystemic shunt ratio which is the amount of portal blood that has shunted away from the liver directly into the systemic circulation. Our patient had a milder form of the intrahepatic shunt with a shunt ratio less than 60% and therefore conservative treatment was chosen while in those with shunt ratios greater than 60% surgery is the optimal intervention [7].

In the pediatric population, patients less than one year of age are generally monitored clinically, as most of the time these portosystemic shunts close after the first year of life. For those who are symptomatic afterward a trial of lactulose and non-absorbable antibiotic therapy is indicated [8]. If medical management does not resolve the symptoms, then intervention should be considered. The first intervention to consider is a radiological intervention such as endovascular occlusion [3,6-7]. If radiological intervention is unsuccessful then surgical ligation should be considered. Liver resection is typically reserved for patients with large multifocal intrahepatic shunts or when none of the above therapies work [1,7-8]. Finally, liver transplantation is a last resort and is almost always carried out on those patients with type 1 extrahepatic shunt after they have failed the occlusion trial or those with multifocal shunt who have developed liver tumor [3].

Other treatment modalities such as endovascular embolization of portosystemic shunts have been shown to be more effective in patients with type 2 extrahepatic shunts or with intrahepatic shunts without underlying cirrhosis. Since there is a direct correlation between age and the diameter of the shunt it has been argued that an intervention should be performed as soon as possible though there are no specific guidelines addressing procedural timing.

Fortunately, our patient did not have any further encephalopathy episodes and was managed medically with lactulose titration. An interdisciplinary team discussion concluded that the best plan of action would be to refer her for shunt closure only in case of any recurrent episodes. The case of our older patient is somewhat unique in that despite the degree and size of her portosystemic shunt she had not developed any symptoms or complications up until this point in her life.

(Abstract: Alvi A, Gupta S, Goyal P, Pichardo J, Mensah E. An Interesting Case of Intrahepatic Porto-Hepatic Shunt as a Cause of Unexplained Encephalopathy. ACG Annual Meeting Abstracts; 2019)

## Conclusions

In summary, the diagnosis of the portosystemic shunts is often challenging and requires a high degree of clinical suspicion. Vascular malformation as a cause of unexplained encephalopathy should always be considered especially in patients with no underling liver cirrhosis, which was the case with our patient. The size of such shunts increase with age and older people are more sensitive to the effect of toxic metabolites which leads to late presentation with these patients. There are various options to treat these patients based on the type and size of shunt, severity of symptoms and comorbidities. These options include an appropriate bowel regimen with the titration of lactulose, non-absorbable antibiotics, endovascular shunt closure or liver transplantation.
